# Differential expression of antennal chemosensory genes related to host preference of *Culex pipiens* biotypes

**DOI:** 10.1186/s13071-025-07028-y

**Published:** 2025-09-23

**Authors:** Rohan Menon, Rickard Ignell, Sharon R. Hill

**Affiliations:** 1 Unit of Chemical Ecology, Department of Plant Protection Biology, Swedish University of Agricultural Sciences, Alnarp, Sweden; 2 Max Planck Center next Generation Insect Chemical Ecology, Alnarp, Sweden

**Keywords:** *Culex pipiens*, Biotypes, Host preference, Antennal transcriptome, Chemosensory genes, Odorant receptors

## Abstract

**Background:**

The northern house mosquito, *Culex pipiens,* is a noted arboviral disease vector commonly found throughout Europe and North America. Two morphologically identical biotypes of this species, *Culex pipiens pipiens* and *Culex pipiens molestus*, display differential host preference to birds and humans, respectively; however, little is known about the genetic mechanisms regulating this behavior.

**Methods:**

Using a Y-tube olfactometer, the host preference of the host-seeking female mosquitoes of both biotypes was tested by providing a choice between synthetic chicken and human odor blends, across 2 days of testing. Antennal transcriptomes, from the mosquitoes that demonstrated a clear and consistent preference to either of the odor blends, were created to observe differences in antennal chemosensory gene expression.

**Results:**

In the host preference experiments, *Cx. pipiens pipiens* and *Cx. pipiens molestus* demonstrated a weak, but significant, preference to the synthetic chicken and human odor blends, respectively, when tested across multiple days. The transcriptome created from the antennae of mosquitoes that made a consistent choice over 2 days of testing identified 9 odorant receptors, 3 ionotropic receptors, and 12 odorant binding proteins, and other chemosensory genes, that were differentially expressed between the two biotypes, which correlate with the observed differential host preference.

**Conclusions:**

This study identified a set of chemosensory genes that are putatively correlated with the differential host preference of the two biotypes. Future research is required to increase the understanding of the function of the identified chemosensory receptors, and how they can be used as genetic markers of host preference of wild mosquitoes.

**Graphical abstract:**

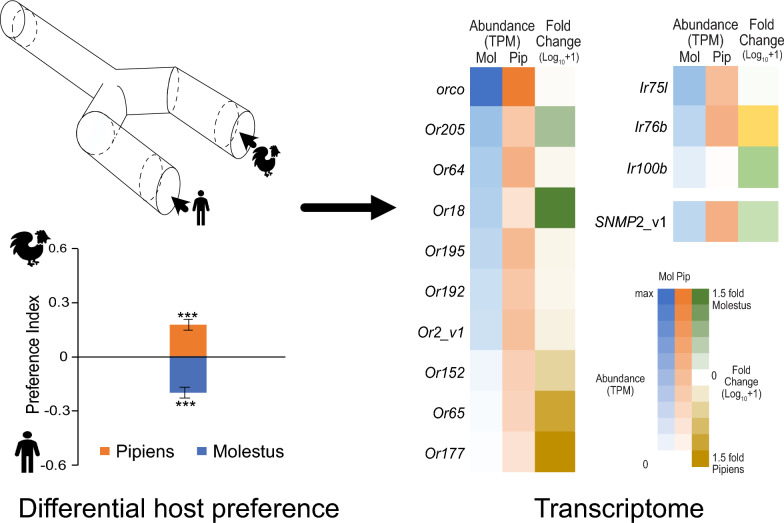

**Supplementary information:**

The online version contains supplementary material available at 10.1186/s13071-025-07028-y.

## Background

The northern house mosquito, *Culex pipiens,* is a vector for arboviruses, e.g., West Nile and Usutu viruses, and is commonly found throughout North America and Europe [1–3]. Two biotypes of the species have been identified: *Culex pipiens* f. *pipiens* (hereafter called Pipiens) and *Culex pipiens* f. *molestus* (hereafter called Molestus), which are morphologically indistinguishable, but differ in terms of ecology, mating, gonotrophic cycle, oviposition site preference, and more notably host preference, with Pipiens and Molestus predominantly feeding on birds (ornithophilic) and humans (anthropophillic), respectively [2]. Although the mechanism regulating the differential host preference is currently unknown, an increased understanding of the genes, and variants thereof, associated with host preference may identify novel targets for vector control, and may be used to create better predictive tools for determining factors regulating vectorial capacity.

Host preference in blood-feeding mosquitoes is defined as the process by which mosquitoes preferentially select one host over others when presented with equal access, while host choice can be defined as the process of detection and feeding on any available host present in the environment [4, 5]. While the factors regulating host preference of the highly anthropophillic vectors of dengue and malaria, *Aedes aegypti and Anopheles gambiae*, respectively, have been well-characterized [5–9], the host preference of *Cx. pipiens* has received less attention [10, 11]. Blood meal analysis, indicating host choice of field-captured Pipiens and Molestus, demonstrates feeding patterns on birds, as well as human and nonhuman mammals, across Europe, which is likely affected by the availability of hosts and trapping location [2, 12–14]. Laboratory studies, however, support a mostly ornithophilic preference of Pipiens [10–12], while Molestus demonstrates a stronger anthropophillic preference [2, 10–12, 15]. These studies, similar to those conducted on other mosquitoes, emphasize that mosquitoes predominantly use olfaction for host discrimination and selection.

Host odors comprise blends of volatile organic compounds (VOCs), which regulate host discrimination and selection [4, 5, 16]. Attraction of blood-seeking mosquitoes to a host is regulated by species-specific differences in detection of odor composition and ratios, with certain classes of chemical compounds, including aldehydes and ketones, being shared across host odors [4, 7, 9]. Mosquitoes may use these shared compound classes for intra- and interspecific host discrimination [4, 7, 9]. For example, differences in the composition of short- and long-chained aldehydes, as well as sulcatone, are thought to regulate interspecific host preference in *Ae. aegypti* [7, 9]*.* In addition, taxon-specific VOCs, such as (*R*)-1-octen-3-ol, have been demonstrated to regulate both intra- and interspecific host discrimination [7, 9, 17].

Volatile organic compounds associated with vertebrate hosts are detected by the peripheral olfactory system of the mosquito, which comprise the antennae, maxillary palps, and labellum [4, 18]. Odorants enter via pores in the hair-like sensilla on these olfactory organs, and are transported through the aqueous sensillum lymph by soluble proteins, including odorant binding proteins (OBPs) and chemosensory proteins (Csps), to the chemosensory receptors on the olfactory sensory neuron dendrites [19–21]. The chemosensory receptors include odorant receptors (Ors), ionotropic receptors (Irs), and gustatory receptors (Grs), with other membrane-bound proteins, such as sensory neuron membrane proteins (SNMPs), also being involved in signal transduction [4, 19, 20]. Both Ors and Irs are heteromeric proteins constituted of a coreceptor, orco, as well as Ir8a, Ir25a, and Ir76b, respectively [19–21]. The Or pathway is sufficient for eliciting host seeking, while both the Or and Ir pathways play a role in host discrimination [22–24]. While the majority of Grs are involved in taste, a subset of Grs is associated with the detection of CO_2_ required for activation and attraction of host-seeking mosquitoes [4, 25–27]. Differential expression of chemosensory genes in the peripheral olfactory system, predominantly *Or*s*,* and functional characterization of sequence variants, in anthropophillic and zoophilic mosquito subspecies and species, furthermore demonstrate a correlation with host preference [7, 8, 28]. While a genome-wide association study identified select Ors correlating with host preference in the two *Cx. pipiens* biotypes [12], there is a lack of studies linking the host preference phenotype with differential chemosensory gene expression in the peripheral olfactory system of Pipiens and Molestus.

The aim of this study was to identify differential expression of chemosensory genes correlating with host preference of Pipiens and Molestus. For this purpose, a two-choice assay was used to assess a consistent host preference of the two biotypes to either synthetic chicken or human odor blends [15, 24]. An antennal transcriptome was obtained from mosquitoes that displayed a consistent preference, to identify differentially expressed genes. The identification of differentially expressed chemosensory genes may provide an insight into the molecular mechanisms regulating the host preference of *Cx. pipiens* biotypes.

## Methods

### Mosquito rearing

Eggs of Pipiens and Molestus were provided in October 2021 by Prof. Sander Koenraadt (Wageningen University, Netherlands), and were reared from colonies established at Wageningen University in 2016, from field collected mosquitoes [29]. Larvae and adult Pipiens and Molestus were reared at 27 ± 2 °C, 65 ± 2% relative humidity with a 12 h light: 12 h dark photoperiod, with the light: dark cycle chosen to mimic the natural light conditions in Europe at times of the year with the highest incidence of West Nile virus transmission (August–September) [30]. Moreover, these rearing conditions have been used in previous experiments aimed at assessing the odor-mediated response of Molestus [15]. Eggs of each biotype were placed in plastic trays (23.5 cm × 18 cm × 7.5 cm) filled with 1 L tap water and fed with Tetramin^®^ fish food (Tetramin, Blacksburg, Germany), with approximately 150–200 larvae per tray. Pupae were collected in water-filled 30 mL plastic cups and placed in Bugdorm-4E1515 cages (17.5 cm × 17.5 cm × 17.5 cm, Megaview Science Co., Taichung, Taiwan) with ad libitum access to 10% sucrose in glass vials with filter paper wicks. Molestus were maintained on a sucrose diet alone, whereas female Pipiens females were provided defibrinated cow blood (Håtunalab, Bro, Sweden) via a Hemotek membrane feeding system (Hemotek Ltd, Blackburn, UK). Molestus adults were allowed to complete their first gonotrophic cycle (4–5 days post-emergence (dpe)), and were tested at peak host-seeking conditions (8 dpe) in accordance with Spanoudis et al. [15]. Pipiens adults used for experiments were not given access to a blood meal and were tested at 4 dpe, concordant with the activity period of Molestus. All mosquitoes were starved with access to water 24 h prior to experimentation and tested at peak host-seeking time (Zeitgeber time 15 ± 2 h) [15].

### Behavioral analysis

A Y-tube olfactometer [15, 24] (120 cm × 10 cm), illuminated from above with red light at 40 lx, was used to assess the host seeking of the two biotypes. Air was passed through a charcoal filter and humidified before entering the olfactometer at 0.3 m s^−1^, with room conditions mimicking rearing conditions (27 ± 2 °C, 70 ± 5% relative humidity). Synthetic host odor blends for human and chicken were made as previously described [15, 24] (Supplementary Table S1). The stock concentration of the odor blends was diluted in pentane (≥ 95%, Carlo Erba Reagents, Emmendingen, Germany) and released by diffusion from wick dispensers to control for a consistent release of all components of the odor blend throughout the behavioral assay [15, 24]. The wick dispensers were placed in glass wash bottles (250 mL; Lenz Laborglas, Wertheim, Germany) and the odors or solvent control were delivered into the upwind end of either arm of the olfactometer via Teflon™ tubing.

Groups of five mosquitoes were placed in cylindrical release cages (10 cm × 10 cm) for 2 h to acclimatize to room conditions. The release cages were then placed downwind of the olfactometer, and the mosquitoes were allowed 5 min to acclimatize, before the odor blend(s) and/or solvent control were introduced into the upwind ends of either arm of the olfactometer. The door of the release cages was opened, and the mosquitoes were given 5 min to make a choice between the two arms. Mosquitoes that did not leave the release cage or that remained within the downwind tube prior to the arms were considered nonresponding, and were excluded from further analyses. A preference index, calculated by (*T* − *C*)/(*T* + *C*), where *T* is the number of mosquitoes responding to the test odor, i.e., chicken odor for Pipiens and human odor for Molestus, and *C* is the number of mosquitoes responding to the other odor tested, was used to determine the host preference of the mosquitoes.

To test differences in host preference of the two biotypes, three assays were conducted using the Y-tube olfactometer. Initially, two pilot experiments were conducted to (a) identify the dose-dependent response to the host odor blends versus a pentane control, and (b) to identify the dose-dependent preference to either odor blend. The purpose of the latter experiment was to identify a dose at which the two biotypes displayed a clear differential host preference. The main experiment (c) was designed to assess whether the biotypes demonstrate a consistency in host preference over time. For this, mosquitoes were provided with a choice between the chicken and human odor blend, using a dose (10^−5^) that elicited a clear differential host response in (b). Pipiens and Molestus that demonstrated a preference to either chicken or human odor, respectively, were collected into Bugdorm cages with ad libitum access to water, and then the assay was repeated 24 h later. Mosquitoes that made a consistent choice over the 2 days were used for subsequent antennal transcriptomic analyses. Three replicates of individuals that displayed a consistency in preference, including mosquitoes of different cohorts for both biotypes, were conducted to obtain 50 individuals for each replicate, which were then subjected to tissue dissection.

### Tissue dissection and RNA extraction

The antennae of cold-anesthetized adult females were collected using sterilized forceps, immediately (< 2 h) after the behavioral assays were completed, and rapidly transferred into RNAlater^®^  (Thermo Fisher Scientific, Stockholm, Sweden), stored at room temperature overnight, and then stored at −20 °C until RNA extraction. Four biological replicates of 50 pairs of antennae per biotype were generated. For RNA extraction, RNAlater was removed and the antennal tissue was disrupted and homogenized using a power pestle with a disposable RNAse-free plastic pestle. Total RNA extraction and DNAse digestion were performed using the RNeasy Mini Kit (Qiagen, Hilden, Germany) following the manufacturer’s protocol and then stored at −80 °C. Prior to sequencing, the RNA quality and quantity were analyzed using a TapeStation system 1200 (Agilent Technologies, Stockholm, Sweden).

### Sequencing and RNA-seq analysis

The eight total antennal RNA samples were shipped on dry ice to Eurofins Genomics (Ebersberg, Germany). Of the eight replicates sent for sequencing, six replicates met the total RNA-seq requirements from Eurofins (three each from Molestus and Pipiens). Sample libraries were constructed using the INVIEW Transcriptome Ultra Low workflow (Eurofins Genomics), which generated paired-end reads of 2 × 150 bp coverage with a depth of 20 Mb. Raw read data was cleaned and trimmed to remove adaptors, and sequences with a Phred score ≤ 20 were discarded using CLC Genomics Workbench (http://www.clcbio.com, version 23.0.5; Qiagen, Vedbæk, DK). Cleaned sequences were mapped to the *Cx. quinquefasciatus* reference genome from VectorBase (*Culex quinquefasciatus* JHB2020, VectorBase rel. 66, 28-NOV-2023) (Supplementary File S1). Owing to discrepancies between the new (JHB 2020) and previous reference genome (Johannesburg) [31], the annotations for the chemosensory gene families in the previous genome were correlated with that in the most recent genome (Supplementary Table S2).

### Differential gene expression analysis

All RNA-seq analyses were performed using CLC Genomics WorkBench. To visualize differential antennal transcript abundance between the two biotypes, the trimmed mean of the M value (TMM) adjusted counts per million, i.e., TPM, for each replicate were calculated. The threshold of 0.6 TPM was chosen following the rationale that this is a reasonable approximation of thresholds used with other normalization methods (i.e., 1 RPKM; 1 FPKM). A gene ontology (GO) analysis was performed on transcripts to confirm the expected expression of functional gene ontologies in the antennae of Pipiens and Molestus, and to observe differences in gene expression between the biotypes associated with host preference, similar to other antennal transcriptomic studies [32, 33]. The GO analyses were performed on transcripts showing significant expression in the transcript libraries, as well as on the genes that were differentially expressed between the two biotypes (FDR *P* ≤ 0.05, fold change ≥ 1.5), using the Vectorbase reference genome annotations stated above. Heat maps were generated by comparing the Log_10_ average TPM for the library of each biotype alongside the FC to compare expression between the biotypes*.*

### Statistical analyses

The behavioral response to the synthetic chicken and human odor blends was analyzed using a beta binomial model followed by Tukey’s multiple comparisons post-hoc test with R software (version 4.3.1) using the packages “readxl,” “emmeans,” and “car.” A beta binomial model was chosen to account for the group of mosquitoes being flown per replicate, using the following formulae:

$$\begin{gathered} {\text{Yij }} = {\text{ BetaBin}}\left( {\mu {\text{i}}} \right) \, \\ {\text{ logit}}\left( {\mu {\text{i}}} \right) \, = \, \alpha {\text{i }} \\ \end{gathered}$$

,in which *i* denotes the blend, with *i* = 1 being the blend the moquitoes choose, and *i* = 2 being the other blend, *j* denotes the *j*th replicate in treatment *i*, *μ*_*i*_ denotes the mean of treatment *i*, *α*_*i*_ denotes the logit-transformed mean, and *Y*_*ij*_ is the success probability. The multiday assays of the biotypes were analyzed using a one-way analysis of variance (ANOVA) to determine the difference in response of mosquitoes over the 2 days of testing. The FDR *P*-values were determined in CLC Genomics Workbench [34].

## Results

### Behavior

**Fig. 1:**
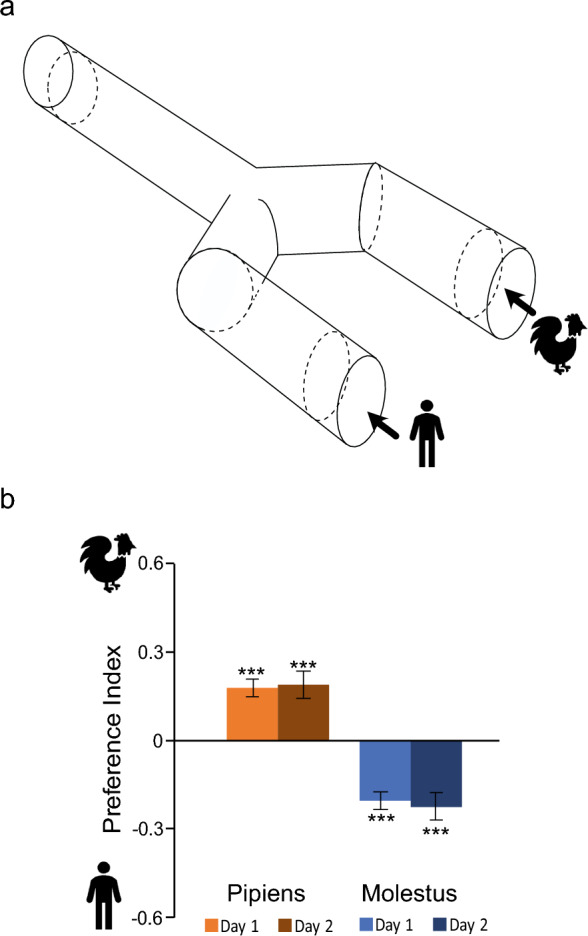
Each of the two biotypes of *Culex pipiens*, Pipiens and Molestus, demonstrate a low but consistent preference to synthetic chicken and human odor blends, respectively. **a** Diagram of the Y-tube olfactometer used to assess host preference of the biotypes. **b** Consistency in host preference for each biotype was assessed over 2  days, by the response to synthetic odor blends (dose = 10^−5^). Error bars represent the standard error of proportions, with asterisks denoting statistical difference from 0 (*P* < 0.0001) for each day

When assayed in pilot Y-tube olfactometer assays (Fig. 1a), female host-seeking Pipiens and Molestus demonstrated a differential dose-dependent behavioral response when presented with a choice between either the synthetic chicken or human odor blends and a solvent control (Supplementary Fig. S1a and b), as well as between the two blends (Supplementary Fig. S1c). Pipiens and Molestus responded to lower doses of the synthetic chicken and human odor blends, when compared with the other biotype, respectively (N= 7–8, *n* = 50), when choosing between either of the odor blends versus the solvent control (Supplementary Fig. S1a and b). For the two-choice experiments, 982 Pipiens and 886 Molestus were tested, with 773 (79%) Pipiens and 759 (86%) Molestus responding to any of the two odor blends. In a choice between the two odor blends (Supplementary Fig. S1c), Pipiens showed a preference to the chicken odor blend at lower doses, which shifted to the human odor blend at the highest dose tested (*F* = 3.07, *df *= 31, *P* = 0.04). In contrast, Molestus showed a dose-dependent preference to the human odor blend. To assess the consistency in preference over time, the two-choice behavioral assay was repeated using the dose eliciting a clear differential host preference in both biotypes. Both biotypes maintained a similar ratio of host preference over 2 days, with Pipiens and Molestus significantly preferring the synthetic chicken (*z* = 4.832, *P* < 0.001) and human (*z* = −5.992, *P* < 0.001) odor blends, respectively (Fig. 1b). Females that demonstrated consistent host odor preference were subsequently used for tissue collection.

### RNA sequencing

**Fig. 2:**
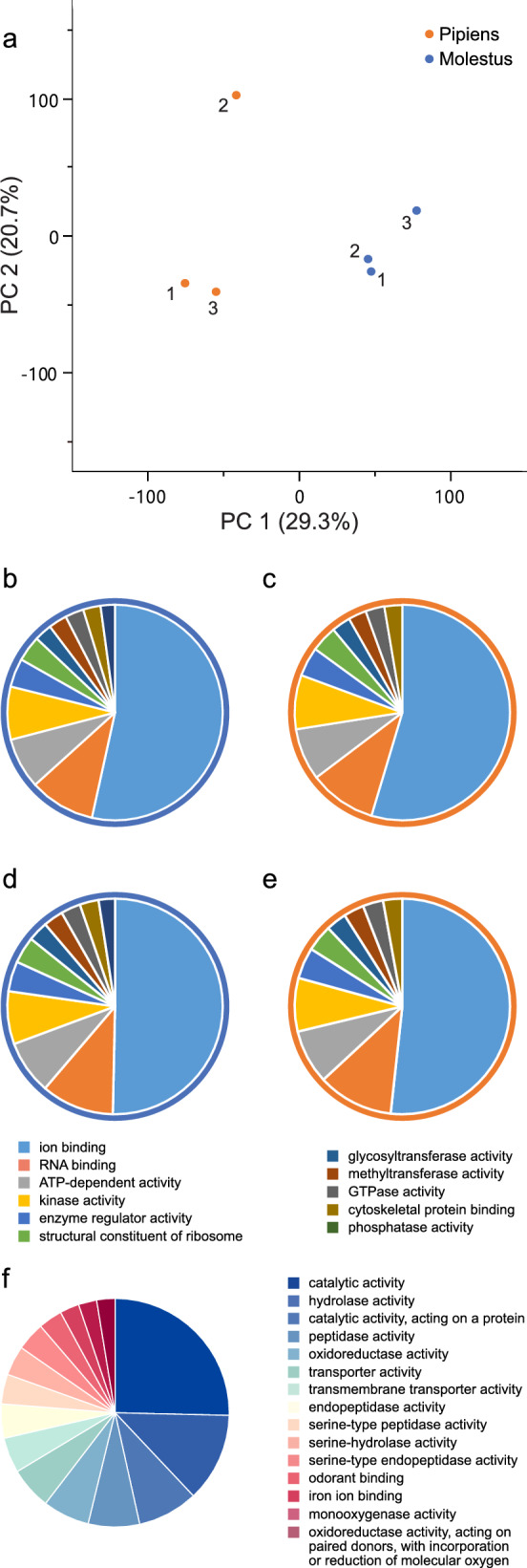
Gene expression and function differ between antennal libraries of host-seeking Pipiens and Molestus. **a** Principal component analysis of the six sample libraries, collected from antennal tissue of the Pipiens and Molestus females that displayed consistent host preference to synthetic chicken and human odor blends, respectively, revealed a clear separation of biotypes along principal component (PC) 1. **b–e** Gene ontology (GO) analysis, using GO slim terms of the genes identified in the transcriptome analysis, demonstrated differences in function of genes in the antennae compared with the background (**b** and **c**) and of genes showing reliable expression (**d** and **e**) in the antennae of Molestus and Pipiens, respectively. Charts with blue borders (**b** and **d**) refer to Molestus, while charts with orange borders refer to Pipiens (**c** and **e**). **f** Gene ontology terms, which were identified when comparing differentially expressed genes between biotypes. The legends (**b–f**) indicate terms representing ≥ 5% of the total expressed transcripts for all comparisons

Expression profiling of antennal total RNA from the six libraries (three each from Molestus and Pipiens), constructed from paired-end reads of 2 × 150 bp coverage with a depth of 20 Mb, showed a similar average level of reliably expressed genes of 10,067 and 10,166 (transcripts per kilobase million (TPM) > 0.6) in Molestus and Pipiens, respectively (Supplementary File S2). A core eukaryotic gene (CEG) analysis identified 353 and 352 out of the 361 CEG genes [35] to be reliably expressed (TPM > 0.6) in Pipiens and Molestus, respectively (Supplementary File S2), demonstrating sufficient sequencing depth and coverage of the sample libraries. A principal component analysis among the antennal transcriptomes of the six libraries demonstrated differential expression of antennal genes between the two biotypes along principal component 1, accounting for 29.3% of the variation (Fig. 2a).

### Gene ontology analysis

The gene ontology (GO) slim terms of genes, related to their molecular function, present at background levels (Fig. 2b and c) and of the genes that were reliably expressed in the antennae (Fig. 2d and e) (TPM > 0.6) of Pipiens and Molestus identified few differences. The GO slim terms of the most abundant genes in all comparisons were ion binding (GO:0043167), RNA binding (GO:0003723), and ATP-dependent activity (GO:0140657). The only significant difference in GO slim terms between the two biotypes was the number of genes in the molecular function category hydrolase activity, acting on carbon–nitrogen (but not peptide bonds) (GO:0016810) in the Pipiens library, which represented 3% of the total number of identified genes (data not shown). The most frequent GO terms of differentially expressed genes between the two biotypes (genes with an absolute fold change ≥ 1.5 and a threshold false discovery rate *P*-value of ≤ 0.05) were catalytic activity (GO:0003824), hydrolase activity (GO:0016787), and catalytic activity, acting on a protein (GO:0140096) (Fig. 2f). Odorant binding (GO:0005549) represented 5% of the differentially expressed genes between the two biotypes, and these genes were selected for further expression analysis.

### Differential expression of chemosensory genes

#### Odorant receptors

**Fig. 3:**
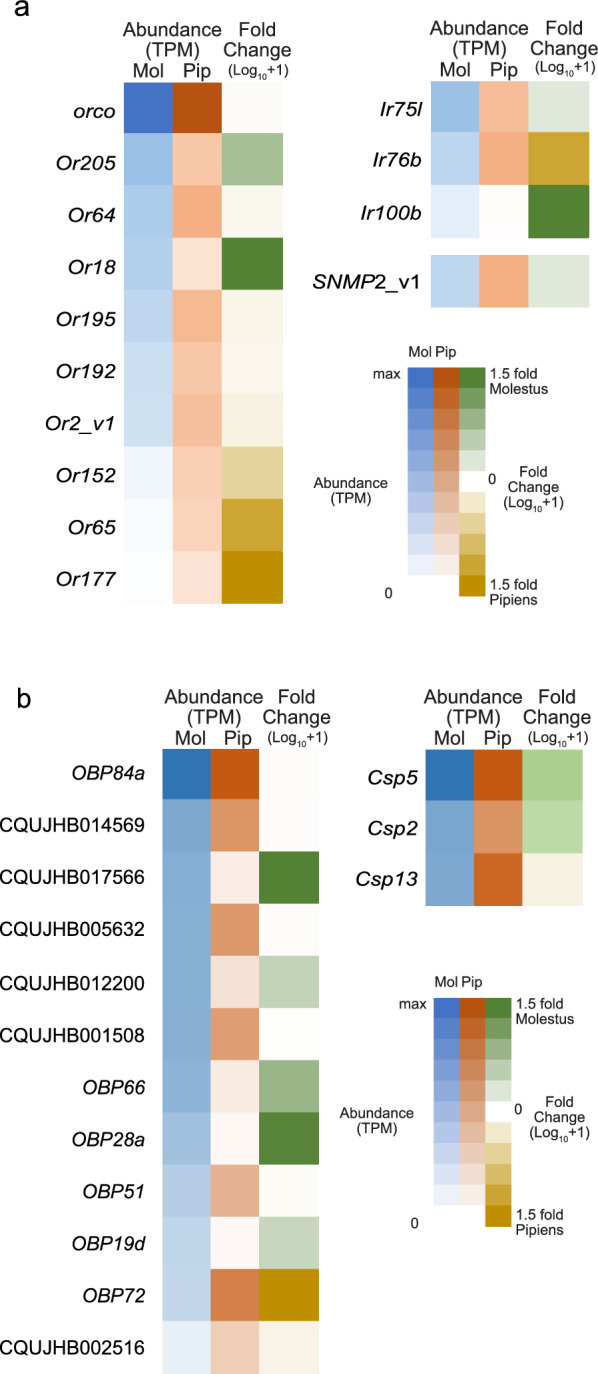
Comparison of differential antennal chemosensory gene expression in Pipiens and Molestus correlated with divergent host preferences. Transcript abundance values represented in Log_10_ + 1 scale for Pipiens (P) and Molestus (M) in orange and blue, respectively, with fold change (FC) representing a comparison between the two biotypes. Genes were labelled as per the reference genome (*Culex quinquefasciatus* JHB2020, VectorBase rel. 66, 2-NOV-2023), and if they were not annotated, the VectorBase gene IDs are stated. **a** Transcript abundance of odorant receptors (*Or*s), ionotropic receptors (*Ir*s), and sensory neuron membrane proteins (*SNMP*s). **b** Transcript abundance for odorant binding proteins (*OBP*s) and chemosensory proteins (*Csp*s)

Of the 156 annotated *Or*s obtained from the reference genome (*Culex quinquefasciatus* JHB2020, VectorBase rel. 66, 28-NOV-2023), 104 and 105 *Or*s were reliably expressed in Pipiens and Molestus, respectively, with the odorant coreceptor *Orco* (CQUJHB017442) being highly expressed in both biotypes (Supplementary File S3). Among the reliably expressed *Or*s, nine were differentially expressed (TPM > 0.6 and fold change > 1.5 or < −1.5), with three: *Or18*, *Or64*, and *Or205* demonstrating higher transcript abundance in Molestus than Pipiens (Fig. 3a). Of the remaining six *Or*s, three, *Or2*, *Or192*, and *Or195,* had higher transcript abundance in Pipiens than Molestus, while *Or65*, *Or152*, and *Or177* were exclusively expressed in Pipiens (Fig. 3a).

#### Ionotropic receptors and other transmembrane chemosensory proteins

Of the 160 annotated *Ir*s obtained from the reference genome, 34 and 40 *Ir*s were reliably expressed in Pipiens and Molestus, respectively, with the *Ir* coreceptors, *Ir8a* (CQUJHB009988), *Ir25a*, and *Ir76b*, being highly expressed in both biotypes (Supplementary File S3). *Ir76b* was the only coreceptor that showed higher abundance in Pipiens than Molestus (Fig. 3a). Out of the variable tuning *Ir*s, two, *Ir75l* and *Ir100b*, had a higher abundance in Molestus than Pipiens (Fig. 3a). Of the 53 annotated *Gr*s, six and ten were reliably expressed in Pipiens and Molestus, respectively, with no *Gr*s being differentially expressed between the two biotypes (Supplementary File S3). Both annotated *SNMP*s were reliably expressed in Pipiens and Molestus (Supplementary File S3), with *SNMP2,* transcript variant  X1 (CQUJHB014288) being more abundant in Molestus than in Pipiens (Fig. 3a).

#### Soluble odorant binding proteins

Of the 88 annotated *OBPs*, 36 and 44 were reliably expressed in Pipiens and Molestus, respectively (Supplementary File S3). A total of 12 *OBP*s were differentially expressed between the biotypes, with three, *OBP66*, CQUJHB012200 and CQUJHB017566, having a higher abundance in Molestus than Pipiens, and *OBP19d* and *OBP28a* being exclusively expressed in Molestus (Fig. 3b). The remaining seven *OBP*s exhibited a higher abundance in Pipiens than in Molestus (Fig. 3b). Of the 22 annotated *Csp*s, 13 and 7 were reliably expressed in Pipiens and Molestus, respectively, with *Csp2* and *Csp5* being more abundant in Molestus than in Pipiens, whereas *Csp13* was more abundant in Pipiens than in Molestus (Fig. 3b).

## Discussion

The two biotypes of *Cx. pipiens* demonstrated a preference for either humans or birds [this study, 10, 12], albeit more variable compared with highly anthropophillic and zoophilic mosquito species [5–9]. An antennal transcriptome created from the *Cx. pipiens* biotypes, demonstrating a consistent host preference, identified differentially regulated chemosensory genes, encoding Ors, Irs and OBPs that confer sensitivity and selectivity to host VOCs, and mediate host seeking and discrimination in mosquitoes [this study, 7, 8]. These genes are targets for future functional characterization aimed at understanding the molecular mechanisms regulating host selection and discrimination of *Cx. pipiens*, which ultimately regulates vectorial capacity.

Pipiens and Molestus demonstrated an ornithophilic and anthropophilic preference, based on their response to the synthetic chicken and human odor blends, respectively, similar to that found in previous studies [10–12]. In a two-choice assay, both biotypes demonstrated a low, however consistent, preference to either host odor, with lower anthropophillic preference of Molestus than previously described [11]. When compared with the highly anthropophillic *Ae. aegypti* and *An. gambiae* [5–7, 36], and the highly zoophilic *Anopheles quadriannulatus* [5, 37]*,* the host preference of Pipiens and Molestus was lower, implying that both biotypes are opportunistic although biased to either humans or birds, which is reflected in blood meal analysis of field caught mosquitoes throughout Europe [13]. Whether the consistently low preference to the synthetic odor blends of the biotypes reflects the low ability of *Culex* mosquitoes to learn odors, compared with strongly anthropophilic species, correlated with differential dopaminergic innervation of the primary olfactory center, the antennal lobe [38], remains to be confirmed. Available data, however, emphasize that Pipiens and Molestus are attracted to, and discriminate between, host odors [this study, 10, 12, 15], which is likely linked to differential expression and sequence variants of chemosensory genes in the primary olfactory organ, the antenna [7, 8]. From an evolutionary perspective, and for closely related taxa, such changes may affect the tuning of OSNs, resulting in a change in preference at a low cost [39, 40].

The Or and Ir pathways mediate host seeking (Ors) and discrimination (Ors and Irs) in mosquitoes [22–24], with other membrane-bound and soluble chemosensory proteins regulating selectivity and sensitivity of the olfactory system [19–21]. In host seeking *Cx. pipiens*, which demonstrated a consistent host preference, three Ors each were more abundant in the antennal transcriptome of Pipiens and Molestus, while three Ors were exclusively expressed in Pipiens, providing a potential molecular mechanism regulating discrimination between humans and birds, similar to that proposed for the anthropophillic and zoophilic subspecies of *Ae. Aegypti* [7]. While the majority of these Ors have not been functionally characterized, Or2 has been shown to bind to indole, 4-methyl phenol, and benzaldehyde, compounds found in human body emanations, but also released by a wide variety of organisms, mainly bacteria, and proposed to mediate host- and oviposition site-seeking [3, 9, 24, 41, 42]. Out of the potential members among the Irs regulating host discrimination, the coreceptor Ir76b and two tuning Irs, were differentially expressed in the two biotypes. Of these, Ir76b and Ir75l mediate responses to carboxylic acids and/or amines, both classes of which are present in human and avian odors [43–46]. *Ir76b* mutant *Ae. aegypti* display a decreased sensitivity and attraction to human odor, while retaining the ability to discriminate between humans, emphasizing a key role of the Or pathway in host discrimination [46]. While the role of OBPs and Csps in regulating mosquito behavior is currently unclear, their role in odorant transport, receptor interaction, and gain control [22], and the differential expression of predominantly OBPs, suggests that these soluble proteins may regulate sensitivity to (select) host VOCs. In summary, the observed differential expression of chemosensory genes provides targets for further functional characterization aimed at understanding the molecular mechanism(s) regulating host preference in *Cx. pipiens* biotypes.

## Conclusions

This study supports the host preference of Pipiens and Molestus, and demonstrated that this preference is innate and not individualistic under laboratory conditions. The transcriptome analyses of expressed antennal genes, including chemosensory genes, in phenotyped female mosquitoes, identified possible molecular mechanisms regulating host preference in the two biotypes. Future research will determine the function of these genes and how they regulate host preference in *Cx. pipiens*, as well as their implication for speciation.

## Supplementary information

Supplementary material 1. Supplementary Table S1. Compounds used for the formulation of the synthetic human and chicken odor blends. Both odor blends used pentane as a solvent. All compounds were obtained from Merck, with purity ranging from 90-99%. Phenol and α-terpineol were obtained as solids, with the amount listed in μg. Supplementary Table S2. List of differentially expressed chemosensory genes identified in the transcriptome analysis, with the corresponding VectorBase gene IDs for the reference genome (*Culex quinquefasciatus* JHB2020, VectorBase rel. 66, 28-NOV-2023) and the corresponding gene IDs from the previous reference genome (*Culex quinquefasciatus* Johannesburg, VectorBase rel. 66)

Supplementary material 2. Supplementary Figure S1. Differential responses of Pipiens and Molestus to the synthetic odor blends and solvent control in a Y-tube olfactometer. Error bars show standard error of proportions, and the preference index (PI) was calculated as PI = (*T − C*)/(*T *+ *C*) where T is the number of mosquitoes responding to the odor blend and C is the number of mosquitoes responding to the solvent control. Mosquito responses to the synthetic human odor blend (**a**) and chicken odor (**b**) against a solvent control (pentane) showed higher sensitivity to the human and chicken odor blends for Molestus and Pipiens, respectively. **c** Dose-dependent responses of Pipiens and Molestus when presented with a choice between the synthetic odors. N= 7 − 8, *n* = 50 for each assay

Supplementary material 3. Supplementary File S1. Total number of reads, mapped reads (%), transcripts and reliably expressed transcripts (>0.6 TPM) of antennal transcriptomes, obtained from behaviorally phenotyped Pipiens and Molestus females

Supplementary material 4. Supplementary File S2. Gene expression data from antennal transcripts of Pipiens and Molestus showing differential host preference. Sheets include all antennal transcripts (Sheet – Antennal genes), genes used for CEG analysis (Sheet – CEG genes), as well as all chemosensory gene families (Sheets – *Or*s, *Ir*s, *Gr*s, *SNMP*s, *OBP*s, *Csp*s). Gene names and VectorBase gene IDs are provided as per the *Culex quinquefasciatus* reference genome (*Culex quinquefasciatus* JHB2020, VectorBase rel. 66, 28-NOV-2023). All expression values are expressed in transcripts per million (TPM) and are color coded for Pipiens (orange) and Molestus (blue). Fold change and false discovery rate (FDR) *P*-values are provided. For the chemosensory genes, heat maps showing expression differences between the two biotypes are provided

Supplementary material 5. Supplementary File S3. Gene expression data from the differentially expressed chemosensory genes of Pipiens and Molestus that showed differential host preference. Gene names, as well as VectorBase gene annotations and IDs, are provided for each gene family. All expression values are expressed in transcripts per million (TPM) and are color coded for Pipiens (orange) or Molestus (blue). Fold change and false discovery rate (FDR) *P*-values are provided. For the chemosensory genes, heat maps showing expression differences between the two biotypes are provided

## Data Availability

All raw sequence data obtained by RNA-seq analysis were deposited in the National Center for Biotechnological Information (NCBI) database under BioProject accession number PRJNA1197149. RNA-seq reads for Molestus 1-3 and Pipiens 1-3 samples are found under the BioSample accession numbers SAMN45774340, SAMN45774341, SAMN45774342, SAMN45774343, SAMN45774344, and SAMN45774345, respectively, in the NCBI database.

## Abbreviations

VOCs: Volatile organic compounds
OBPs: Odorant binding proteins
Csps: Chemosensory proteins
Ors: Odorant receptors
Irs: Ionotropic receptors
Grs: Gustatory receptors
SNMPs: Sensory neuron membrane proteins
TPM: Transcripts per million
GO: Gene ontology
