# Supplementary figures and images for: Differential expression of antennal chemosensory genes related to host preference of Culex pipiens biotypes
Source: Parasit Vectors. 2025 Sep 23;18:372. doi: 10.1186/s13071-025-07028-y (PMC12459065; doi:10.1186/s13071-025-07028-y)

# a Human blend vs Pentane

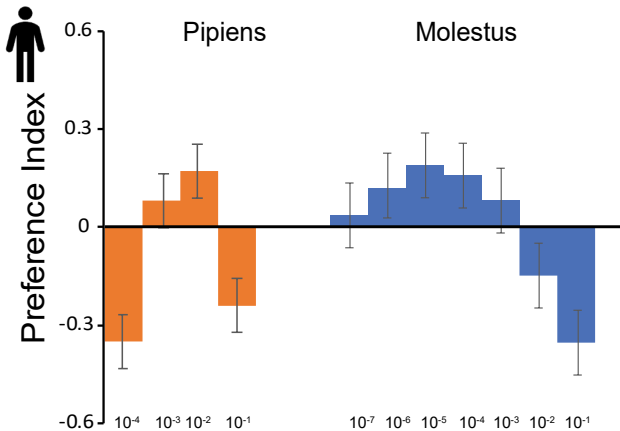

# b Chicken blend vs Pentane

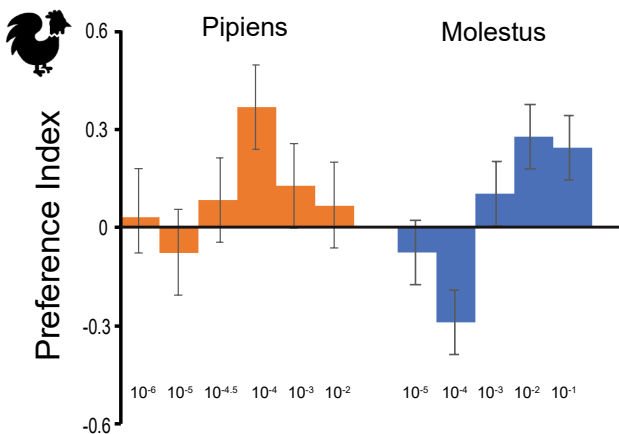

# c Chicken blend vs Human blend

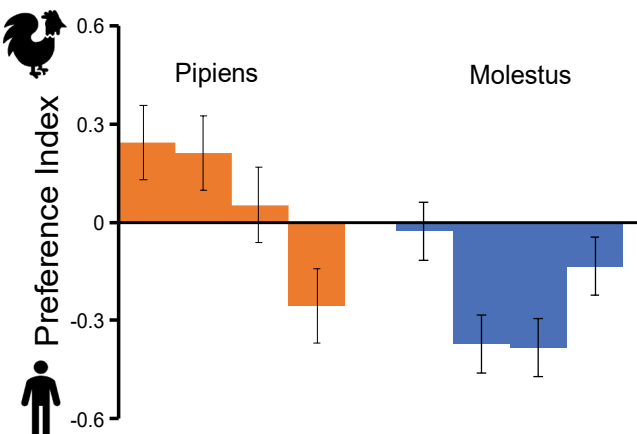

Supplement: Supplementary file 2 — Supplementary material 2. Supplementary Figure S1. Differential responses of Pipiens and Molestus to the synthetic odor blends and solvent control in a Y-tube olfactometer. Error bars show standard error of proportions, and the preference index (PI) was calculated as PI = (T − C)/(T + C) where T is the number of mosquitoes responding to the odor blend and C is the number of mosquitoes responding to the solvent control. Mosquito responses to the synthetic human odor blend (a) and chicken odor (b) against a solvent control (pentane) showed higher sensitivity to the human and chicken odor blends for Molestus and Pipiens, respectively. c Dose-dependent responses of Pipiens and Molestus when presented with a choice between the synthetic odors. N= 7 − 8, n = 50 for each assay [file 13071_2025_7028_MOESM2_ESM.pdf]
